# Assessment of hypermucoviscosity as a virulence factor for experimental *Klebsiella pneumoniae *infections: comparative virulence analysis with hypermucoviscosity-negative strain

**DOI:** 10.1186/1471-2180-11-50

**Published:** 2011-03-08

**Authors:** Yi-Chun Lin, Min-Chi Lu, Hui-Ling Tang, Hsu-Chung Liu, Ching-Hsien Chen, Keh-Sen Liu, Chingju Lin, Chien-Shun Chiou, Ming-Ko Chiang, Chuan-Mu Chen, Yi-Chyi Lai

**Affiliations:** 1 Department of Life Sciences, National Chung-Hsing University, Taichung, Taiwan; 2 Division of Infectious Diseases, Department of Internal Medicine, Chung-Shan Medical University Hospital, Taichung, Taiwan; 3 Department of Microbiology and Immunology, Chung-Shan Medical University, Taichung City, Taiwan; 4 Department of Veterinary Medicine, National Chung-Hsing University, Taichung, Taiwan; 5 Division of Gastroenterology and Hepatology, Department of Internal Medicine, China Medical University Hospital, Taichung, Taiwan; 6 Division of Chest Medicine, Department of Internal Medicine, Chung-Shan Medical University Hospital, Taichung, Taiwan; 7 Infectious Diseases Division, Department of Internal Medicine, St. Joseph's Hospital, Yunlin, Taiwan; 8 Department of Physiology, College of Medicine, China Medical University, Taichung; Taiwan; 9 The Central Branch Office, Center for Disease Control, Department of Health, Taichung, Taiwan; 10 Department of Life Science, National Chung-Cheng University, Chia-Yi, Taiwan

## Abstract

**Background:**

*Klebsiella pneumoniae *displaying the hypermucoviscosity (HV) phenotype are considered more virulent than HV-negative strains. Nevertheless, the emergence of tissue-abscesses-associated HV-negative isolates motivated us to re-evaluate the role of HV-phenotype.

**Results:**

Instead of genetically manipulating the HV-phenotype of *K. pneumoniae*, we selected two clinically isolated K1 strains, 1112 (HV-positive) and 1084 (HV-negative), to avoid possible interference from defects in the capsule. These well-encapsulated strains with similar genetic backgrounds were used for comparative analysis of bacterial virulence in a pneumoniae or a liver abscess model generated in either naïve or diabetic mice. In the pneumonia model, the HV-positive strain 1112 proliferated to higher loads in the lungs and blood of naïve mice, but was less prone to disseminate into the blood of diabetic mice compared to the HV-negative strain 1084. In the liver abscess model, 1084 was as potent as 1112 in inducing liver abscesses in both the naïve and diabetic mice. The 1084-infected diabetic mice were more inclined to develop bacteremia and had a higher mortality rate than those infected by 1112. A mini-Tn*5 *mutant of 1112, isolated due to its loss of HV-phenotype, was avirulent to mice.

**Conclusion:**

These results indicate that the HV-phenotype is required for the virulence of the clinically isolated HV-positive strain 1112. The superior ability of the HV-negative stain 1084 over 1112 to cause bacteremia in diabetic mice suggests that factors other than the HV phenotype were required for the systemic dissemination of *K. pneumoniae *in an immunocompromised setting.

## Background

As a common pathogen responsible for a wide range of clinical illnesses, *K. pneumoniae *has long been the principal cause of pneumonia [1], emerging as the major pathogen associated with pyogenic liver abscesses over the past decade [2]. *K. pneumoniae *has been implicated in 7-12% of hospital-acquired pneumoniae in ICUs in the United States [3,4], accounting for 15, 32, and 34% of community-acquired pneumoniae in Singapore [5], Africa [6], and Taiwan [7], respectively. In the 1990 s, *K. pneumoniae *surpassed *E. coli *as the number one isolate from patients with pyogenic liver abscesses in Taiwan [8], where more than 1,000 cases have been reported [2]. Liver abscesses caused by *K. pneumoniae *(KLA) have become a health problem in Taiwan and continue to be reported in other countries. Metastatic lesions, such as meningitis and endophthalmitis, develop in 10-12% of KLA patients and, worsening the prognosis of this disease [2]. Cases of KLA in Taiwan typically occur in diabetic patients with a prevalence rate from 45% to 75% [9,10].

Diabetes mellitus (DM), the most common endocrine disease, is a predisposing factor for infections of *K. pneumoniae *[9]. Type 1 diabetes (IDDM) is a form of DM resulting from autoimmune triggered destruction of insulin-producing β cells of the pancreas. Type 2 diabetes (NIDDM) is characterized by high blood glucose within the context of insulin resistance and relative insulin deficiency. In 2000, approximately 171 million people in the United States were affected by diabetes, and this number is expected to grow to 366-440 million by 2030 [11]. Diabetes can lead to a variety of sequelae, including retinopathy, nephropathy, neuropathy, and numerous cardiovascular complications, and patients with diabetes are more prone to infection. Several factors predispose diabetic patients to infection, including genetic susceptibility, altered cellular and humoral immune defense mechanisms, poor blood supply, nerve damage, and alterations in metabolism [12].

Clinical *K. pneumoniae *isolates produce significant quantities of capsular polysaccharides (CPS). Several CPS-associated characteristics have been identified in correlation with the occurrence of KLA, including serotype K1 or K2 [13] and a mucopolysaccharide web outside the capsule, also known as the hypermucoviscosity (HV) phenotype [14]. We collected 473 non-repetitive isolates from the foci of *K. pneumoniae-*related infections. Interestingly, the incidence of strains displaying the HV phenotype in the *K. pneumoniae *abscess isolates was 51% (48/94), which was significantly lower than that reported by Yu et al. (29/34, 85%) [15] and Fang et al. (50/53, 98%) [14]. A decline in the HV-positive rate suggests the emergence of etiological HV-negative strains and urges a re-evaluation of whether the HV phenotype acts as a virulence determinant for clinical *K. pneumoniae *isolates. Due to the significant susceptibility of diabetic patients, this study established two infection models recapitulating pneumonia and KLA in diabetic and naïve C57BL/6J mice. The role of the HV phenotype in the pathogenesis of *K. pneumoniae *was determined in these mouse models by comparatively analyzing bacterial virulence for two clinically isolated K1 strains, 1112 and 1084, which were well-encapsulated with similar genetic backgrounds; however, only 1112 exhibited the HV-phenotype.

## Results

### Emergence of HV-negative *K. pneumoniae *related to tissue abscesses

**Figure 1:**
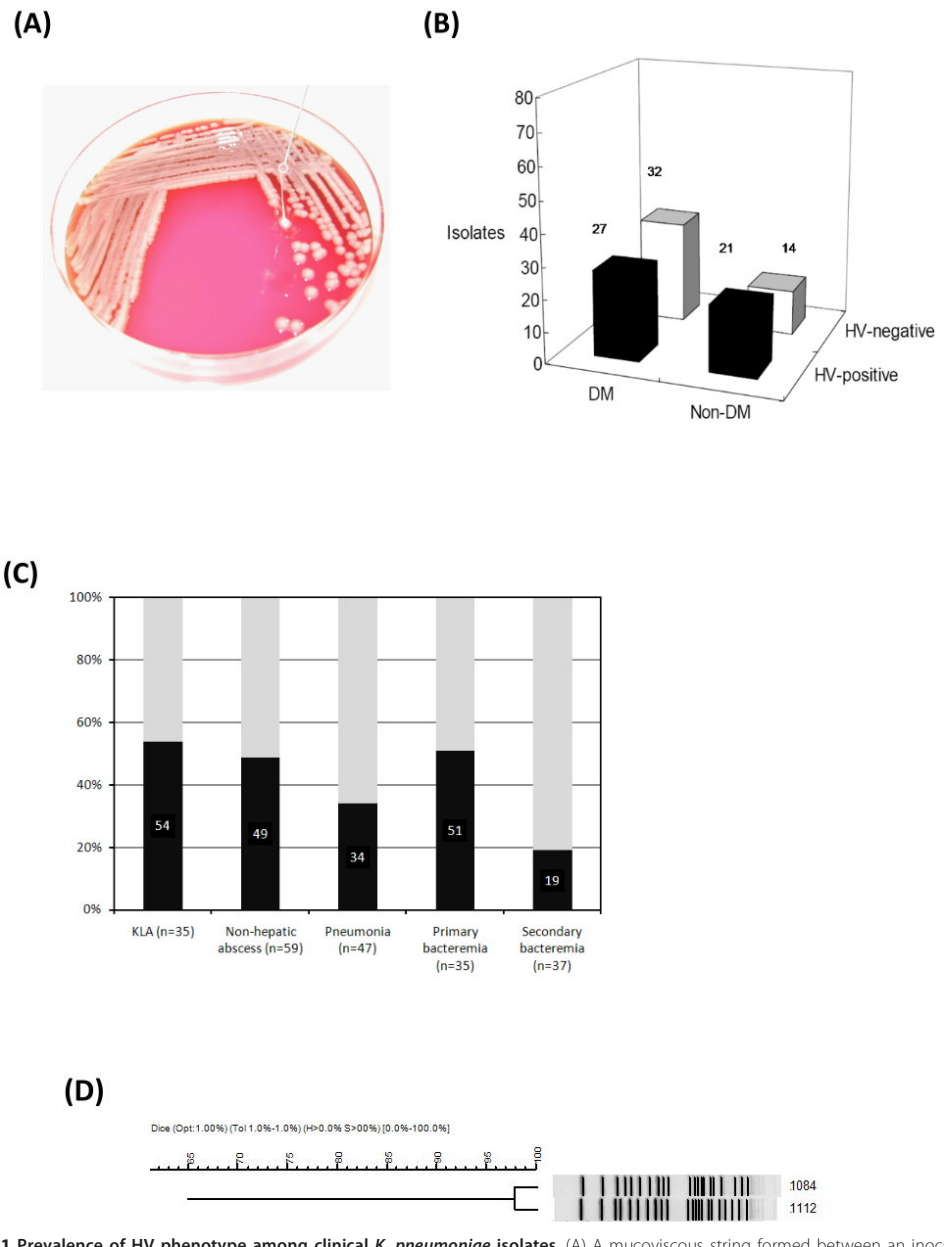
**Prevalence of HV phenotype among clinical *K. pneumoniae *isolates**. (A) A mucoviscous string formed between an inoculation loop and the colony of a HV-positive strain. (B) Occurrence of HV-positive (black columns) or HV-negative (white columns) isolates in patients with or without diabetic mellitus (DM or Non-DM). (C) Prevalence of HV-positive *K. pneumoniae *among patients suffering from various infections, including KLA, non-hepatic abscess, pneumonia, primary bacteremia, and secondary bacteremia. (D) Dendrogram of the HV-positive strain 1112 and-negative strain 1084. Genetic similarities were calculated using UPGMA.

To determine the clinical impact of the HV characteristics, 473 non-repetitive isolates were collected from consecutive patients exhibiting *K. pneumoniae-*related infections under treatment at a referral medical center in central Taiwan, during April 2002-June 2003. Of the clinical isolates, 7% (n = 35) were KLA strains, obtained from tissue-invasive cases presenting the formation of liver abscesses; 13% (n = 59) were isolated from non-hepatic abscesses, including lesions occurring as empyema, endophthalmitis, necrotizing fasciitis, and septic arthritis, as well as lung, epidural, parotid, paraspinal, splenic, renal, prostate, muscle, and deep neck abscesses; 24% (n = 113) were obtained from non-abscess-related cases, including pneumonia without abscess, primary peritonitis, cellulitis, biliary tract infection, primary bacteremia, and catheter-related infections; and 56% (n = 265) were secondary *K. pneumoniae *infections. The HV-phenotype of the 473 strains was determined using the string-forming test (Figure 1A). Interestingly, the HV-positive rate in the tissue-abscess isolates (n = 94) was only 51%, which was significantly lower than that reported by Yu et al. (29/34, 85%) [15] and Fang et al. (50/53, 98%) [14]. In particular, the tissue-abscess isolates from diabetic patients were more frequently HV-negative than those from non-diabetic patients (54% vs. 40%; Figure 1B). Moreover, HV-negative *K. pneumoniae *accounted for the majority of cases related to pneumonia (n = 47; 66%) and secondary bacteremia (n = 37) (Figure 1C). Although HV-negative *K. pneumoniae *are considered less virulent than HV-positive strains, our epidemiological observations indicate that *K. pneumoniae *strains displaying no HV-phenotype have emerged as etiological agents for tissue-abscesses.

### Analysis of comparative virulence for HV-positive and-negative *K. pneumonia*

**Figure 2:**
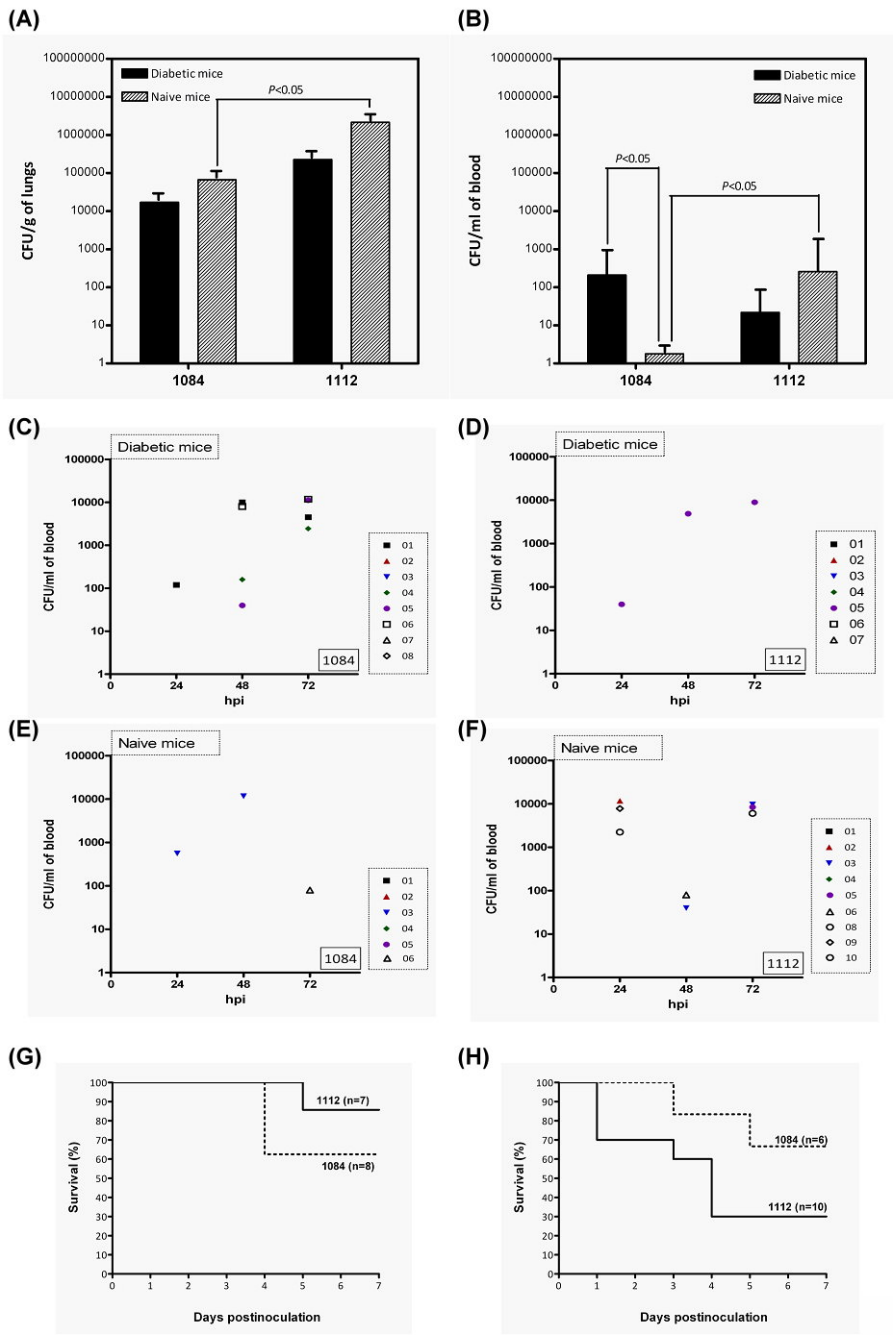
**Analysis of comparative virulence analysis for HV-positive and -negative *K. pneumoniae***. In the pneumonia model, bacterial counts in the lung (A) and blood (B) at 20 hours post-infection with the HV-negative 1084 or the HV-positive 1112 were determined in diabetic mice (filled columns) or naïve mice (striped columns). In the KLA model, 1084 (C, E) and 1112 (D, F) were orally inoculated into diabetic mice with inoculums of 10^5 ^CFU (C, D) or into naïve mice with inoculums of 10^8 ^CFU (E, F). Twenty microliters of blood was removed from the retroorbital sinus of mice at 24 h, 48 h, and 72 h post-inoculation; and the bacterial loads were determined using the plate-counting method. Each symbol represents the data obtained from a particular mouse. The bacterial load recovered from a particular mouse tissue, which was beyond the detection limit (approximately 40 CFU), is not represented. Survival of these mice was monitored daily for seven days. The survival rate of the 1112-infected (solid line) or the 1084-infected (dotted line) diabetic (G) or naïve (H) mice was determined by Kaplan-Meier analysis. Data were compiled from results obtained from three independent experiments.

Under the premise that capsules are unaffected, we selected two clinically isolated K1 strains, 1112 (HV-positive) and 1084 (HV-negative) instead of using genetically manipulated strains to evaluate the role of HV phenotype in *K. pneumoniae *pathogenesis. Both strains were well-encapsulated with the only phenotypic differences in the HV-phenotype, displaying a relatively high genetic identity (>98%) on their PFGE-*Xba*I pulsotypes among the 473 clinical isolates (Figure 1D). Bacterial virulence of the HV-positive strain 1112 and-negative strain 1084 was analyzed comparatively in a pneumoniae or KLA infection model generated in either diabetic or naïve mice. A multi-STZ injection method [16] was used to induce diabetes in mice. The random blood sugar levels of the STZ-treated mice was significantly higher than those of naïve mice at eight weeks (301.86 vs. 123.97 mg/dl, *P *≤ 0.05; Additional file 1:Figure S1A) and thirty weeks (404.36 vs. 121.09 mg/dl, *P *≤ 0.05) post-injection in conjunction with the classical symptoms of polyuria, polydipsia, polyphagia, and hyperglycemia, exhibited in STZ-treated mice, the body weight of the mice was also lowered significantly in a time-dependent manner (Additional file 1: Figure S1B). These results indicate that diabetes was successfully induced in these mice. To recapitulate a pneumonia infection, 30-wk-old diabetic mice or age-matched naïve mice were intratracheally inoculated with 10^4 ^CFU of *K. pneumoniae *1112 (HV-positive) or 1084 (HV-negative). At 20 h post-infection (hpi), 1112 demonstrated a significantly higher proliferation of 1084 in the lungs (Figure 2A, P < 0.05) and blood of naïve mice (Figure 2B, P < 0.05). However, 1084 (the HV-negative strain) had a significant growth advantage in the blood of diabetic mice compared to that of naïve mice (Figure 2B, P < 0.05). This growth advantage of 1084 in the blood of diabetic mice was absent for 1112 (Figure 2B).

**Figure 3:**
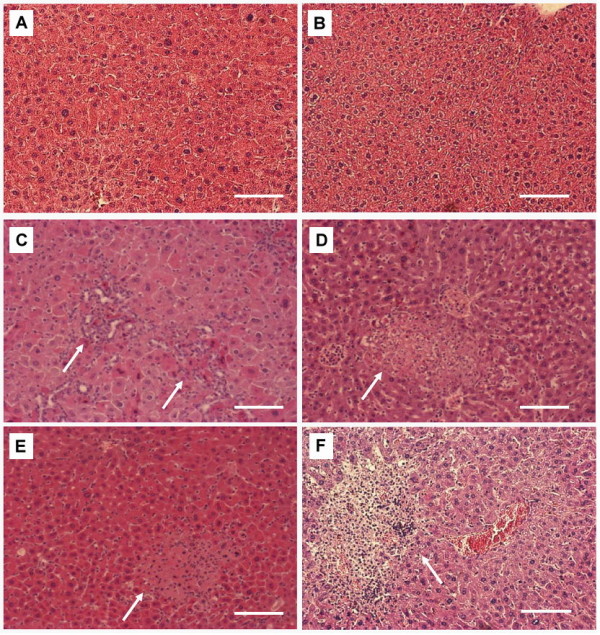
**Histopathological examination of livers**. Mice that had been orally inoculated with PBS (A, B), HV-negative strain 1084 (C, D), or HV-positive strain 1112 (E, F) (in diabetic mice) (A, C, E) with inoculums of 10^5 ^CFU or in naive mice with inoculums of 10^8 ^CFU (B, D, F) were euthanized at seven days post-inoculation. Arrows indicate the area of PMN infiltration and aggregation (100 × magnification). Scale bar represents a distance of 1 μm.

Previous studies have suggested that liver abscesses are caused mostly by HV-positive *K. pneumoniae *[14]. Nevertheless, 46% of our KLA isolates lacked the HV-phenotype, which encouraged us to determine the importance of the HV-phenotype for K1 *K. pneumoniae *in the development of KLA. Based on the KLA model established in our previous study [17], 30-wk-old diabetic or age-matched naïve mice were orally inoculated with 1112 or 1084. Bacterial loads in the blood were determined at 24, 48, and 72 hpi to evaluate the tissue-invasiveness of these strains. Interestingly, 50% (4/8) of the 1084-infected diabetic mice developed bacteremia at 48 hpi with average bacterial load of 4.6 × 10^3 ^CFU/ml (Figure 2C), whereas only 14% (1/7) of the 1112-infected diabetic mice had bacteria in the blood (Figure 2D). The enhanced invasiveness of 1084 contributed to its virulence in diabetic mice, as 37.5% (3/8) of diabetic mice succumbed to 1084 infection, whereas none of the 1112-infected diabetic mice died before day, 4 post-infection (Figure 2G). However, the superior virulence of 1084 over 1112 in diabetic mice was absent in naïve mice. Compared to the presence of 1112 in 70% (7/10) of the infected mice (Figure 2F), 1084 was only detected in the blood of 33.3% (2/6) of the infected naïve mice (Figure 2E). Seven of ten 1112-infected naïve mice died at day 4 but only one of the six 1084-infected naïve mice died at precisely the same time (Figure 2H). Regardless of the HV-phenotype, both 1112 and 1084 induced microabscess foci in the livers at seven days post-inoculation, compared to the control group (Figure 3A, B), as significant infiltrates of polymorphonuclear leukocytes were noted in either the diabetic mice (Figure 3C, E) or the naïve mice (Figure 3 D, F).

### Requirement of HV-phenotype for *K. pneumoniae *1112 virulence

The HV-positive strain, 1112, demonstrated stronger virulence than 1084 in naïve mice. To determine whether the virulence of 1112 was determined by the expression of HV-phenotype, we isolated a mutant that lost its HV-phenotype from a mini-Tn*5 *mutant library of 1112 and designating it KPG6. Based on sequence determination, the mini-Tn*5 *in KPG6 was inserted into the reading frame of *pgi*. Glucose-6-phosphate isomerase, encoded by *pgi*, is one of the key enzymes responsible for exopolysaccharide synthesis of *Klebsiella *[18]. Compared to its parental strain 1112, the HV-negative mutant KPG6 was more sensitive to serum-killing and its virulence in naïve BALB/c mice was significantly attenuated with > 100 fold increase in the oral LD_50 _(Table 1). Additionally, the loss of HV-phenotype also impaired the anti-phagocytosis ability, as the intracellular survival of KPG6 was lower in Raw264.7 macrophages than that of 1112 (Table 1). These results suggest that the HV-phenotype was a virulence determinant for the HV-positive strain 1112.

**Table 1:** Virulence characteristics of *K. pneumoniae *1112, KPG6, and 1084.

Characteristics	*K. pneumoniae *strains		
	
	1112	KPG6	1084
Hypermucoviscosity	Positive	Negative	Negative
Serum killing*^a^*	Resistant	Sensitive	Resistant
Oral LD50*^b^*	6.9 × 10^6 ^	> 10^9^	9 × 10^6^
Intracellular survival (%) in Raw264.7 cell*^c^*	60.29 ± 5.04	46.77 ± 1.61	57.82 ± 2.42

Note. *^a^*If the bacteria examined showed a reduction of 2 log_10 _in the surviving counts after serum incubation, this strain was defined as serum-sensitive. If the viability of a strain remain > 90% after 30 min-treatment, the strain was defined as serum-resistant. *^b^*Five 8 wk old male BALB/c mice of a group were orally inoculated with bacterial culture of a particular *K. pneumoniae *strain in 10-fold steps graded doses. The 50% lethal doses, based on the number of survivors after one week, were calculated by the method of Reed and Muench [29] expressed as colony forming units (CFU). *^c^*A particular *K. pneumoniae *strain was used to infect Raw264.7 cells with m.o.i. = 100. After stringent washes, the number of adherent and intracellular *K. pneumoniae *was determined before and after gentamicin treatment. The intracellular survival rate was calculated as 100% × (number of intracellular bacteria after gentamicin treated for 3 h/number of adherent bacteria before gentamicin treatment).

## Discussion

A capsule-associated mucopolysaccharide web, also known as the hypermucoviscosity (HV) phenotype, was previously considered a characteristic associated with pyogenic *K. pneumoniae *infections [14,15]. Nevertheless, the prevalence of *K. pneumoniae *negative for HV-phenotype in our pyogenic cases (49%; 46/94) suggests that HV-negative strains have emerged as etiologic in the formation of tissue abscesses. HV-negative-associated infections were related to diabetic conditions, as diabetic patients suffering from pyogenic infections were more frequently associated with HV-negative strains than with HV-positive strains (70% vs. 56%). Therefore, in this study, we aimed to assess how essential the HV-phenotype is for *K. pneumoniae *pathogenesis by comparing the virulence of clinically isolated strains that were naturally HV-positive or -negative. Because K1 is the predominant serotype in KLA cases, we selected two K1 strains, 1112 and 1084, which have relatively high genetic similarity among our clinical isolates. Not surprisingly, the HV-positive strain 1112 demonstrated greater virulence than the HV-negative strain 1084 in either a pneumonia or KLA infection model in naïve mice. However, in the KLA model of diabetic mice, 1084 was as potent as 1112 in inducing liver abscesses, exhibiting an ability superior to that of 1112 in causing bacteremia and mortality in mice. The advantageous tissue-invasive ability of 1084 indicates that the HV-phenotype per se is not a determinant for *K. pneumoniae *virulence in a diabetic host.

Genetic loci, including *magA *[14], the *cps *gene cluster [19], the *wb *gene cluster [20], and *rmpA *[21], have been associated with the HV-phenotype. Mutations of these genes have resulted in the loss of the HV-phenotype in conjunction with defects in capsular integrity, confirming the findings of Fang et al. [14], who reported that capsule-related properties, including serum resistance, anti-phagocytosis, and virulence to mice, were drastically attenuated in the *magA *mutants. Ideally, the capsule and HV-phenotype should be investigated independently. However, all of the HV-phenotype-associated genes identified thus far are involved in the regulation or the biosynthesis of capsular polysaccharides. Given that significant quantities of clinically isolated *K. pneumoniae *are well-encapsulated but negative for HV-phenotype, these naturally- selected HV-negative strains could be used as an ideal control for HV-positive strains to minimize the influence of defects on the capsule. Consistent with previous thoughts, the HV-positive strain 1112 was more likely to cause pneumonia or KLA in naïve mice than 1084. Although the idea that the HV-phenotype is a determinant for *K. pneumoniae *virulence was suggested by the fact that the isogenic HV-negative mutant of 1112, KPG6, notably lost its virulence to mice, we could not exclude the possibility that the mutation of *pgi *influenced the integrity of the capsule and disrupted the synthesis of exopolysaccharides as the anti-phagocytic ability of KPG6 in Raw264.7 macrophages was attenuated. Unlike KPG6, naturally-selected HV-negative strain 1084 exhibited the wild-type level capsule-related characteristics, including serum-resistance, anti-phagocytosis, and virulence to mice. The findings suggest that HV-phenotype-related properties are not necessarily the same as the properties related to capsules. Further studies are required to differentiate the roles of the HV-phenotype and capsule in *K. pneumoniae *pathogenesis.

Diabetes is a risk factor for *K. pneumoniae *infections [2,22]. To clarify the role of HV-phenotype in diabetic individuals, we produced diabetes in mice using a STZ-induction method [16]. The STZ-treated diabetic mice were raised to the age of thirty weeks to avoid immunomodifying effects of STZ occurring after administration of the drug [23], to ensure the physiological properties of clinical diabetes occurring in mice, and to mimic middle-aged diabetic persons, the population most susceptible to *K. pneumoniae *infections [2,24]. In pneumonia or the KLA model generated in the diabetic mice, bacteremia was more likely to develop following an intratracheal- or oral-infection with the HV-negative strain 1084 compared to that of 1112. The pathological advantages of HV-negative *K. pneumoniae *in diabetic mice implies that diabetes might provide a specialized environment permitting these strains to disseminate from local tissues, such as the lungs and intestines into the blood. Although previous studies have indicated that the hyperglycemic state of diabetes provokes a functional decline of neutrophils [25,26], phagocytosis by neutrophils from diabetic patients of *K. pneumoniae *1112 was comparable to that of 1084 (data not shown). Moreover, pulmonary infections caused by *K. pneumoniae *1112 and 1084 caused similar apoptosis levels of the alveolar macrophages in both diabetic and naïve mice (data not shown). Given that capsules play a pivotal role in the protection of *K. pneumoniae *from phagocytosis [27], it is not surprising that the well-encapsulated *K. pneumoniae *1084 interacted with phagocytes in the same manner as 1112. This implied that the HV phenotype was not essential for the antiphagocytosis of *K. pneumoniae*. Thus, a mutant library of 1084 generated using a signature-tagged mutagenesis technique is currently under *in vivo *screening in diabetic mice. Identification of the genetic requirement of 1084 with regard to virulence will provide insights into the means by which 1084 gains an advantage in dissemination and proliferation in the blood of diabetic mice. To our knowledge, this is the first study using naturally-selected strains to evaluate the requirements of HV-phenotype for *K. pneumoniae *virulence in diabetic mice. Our findings suggest that the HV-negative strain 1084 is more virulent than the HV-positive strain 1112 under diabetic conditions, the naturally-selected strain 1084 may serve as an ideal model for identifying virulence factors, rather than relying on the HV phenotype that contributes significantly to the pathogenesis of *K. pneumoniae*.

## Conclusions

HV-phenotype is a virulent determinant for clinically isolated HV-positive *K. pneumoniae*. However, factors other than the HV-phenotype contribute significantly to the virulence of *K. pneumoniae *isolates displaying no HV-phenotype, particularly for systemic dissemination under diabetic conditions.

## Methods

### Bacterial isolates

During a fifteen-month period from April 2002, a total of 473 non-repetitive *K. pneumoniae *were isolated from the infection foci of patients who had *K. pneumoniae*-related infections treated at a referral medical center in central Taiwan. The clinical isolates, which were confirmed as *K. pneumoniae *using the API 20E system (BioMerieux), were collected from various infection foci: 11.6% were from blood; 4%, from liver aspirates; 0.4%, from eye aspirates; 0.8%, from cerebrospinal fluid; 26.2%, from non-hepatic abscesses; 22.8%, from sputum; 8.5%, from wound pus; and 25.6%, from other body fluids. Due to the difficulty in determining whether *K. pneumoniae *is the primary pathogen in a urinary tract infection, urine isolates were excluded. If cultures were concomitantly positive in more than one site, only that culture which was isolated from the primary infection focus was included. One isolate per patient was analyzed, and each isolate represented a single case. Isolates were cultured in Luria-Bertani (LB) broth and stored at -80°C until use. Medical records were reviewed and information related to clinical manifestations and underlying diseases was collected. Clinical research was conducted according to the human experimentation guidelines of Chung-Shan Medical University. Ethical approval was not needed for the present study.

### Determination of the hypermucoviscosity (HV) phenotype and detection of HV-related genes

The HV phenotype display was examined with a string-formation test as described by Fang et al [14]. Bacterial strains to be tested were inoculated onto 5% sheep blood plates and incubated at 37°C for 16 h. Positive of hypermucoviscosity phenotype was defined as the formation of viscous strings > 5 mm in length when a standard inoculation loop was used to stretch the colony on blood agar plates. *K. pneumoniae *isolates, capable of displaying the HV-phenotype from three independent tests were described as HV-positive and those that were unqualified in string forming were HV-negative.

### Induction of diabetes in mice

Six-week-old male C57BL/6J mice were purchased from the National Laboratory Animal Center (NLAC, Taiwan) and allowed to acclimatize in the animal house for one week before experiments. Mice (25-30 g body weight) were randomly divided into two groups. One group received intraperitoneal injection of the pancreatic β-cell toxin streptozotocin (STZ; Sigma) for five days (55 mg/kg per day in 0.05 M citrate buffer, pH 4.5) [16]. The other group received injections of citrate buffer as the control. The serum glucose concentrations and body weights of the mice were determined at indicative time points after the multi-injection of STZ.

### Pneumonia or KLA infection models

To recapitulate a pneumonia infection, thirty-week-old mice were anesthetized with isoflurane and intratracheally inoculated with 10^4 ^CFU of *K. pneumoniae *by intubation with a blunt-ended needle [28]. At 20 h post-inoculation, lungs and blood were retrieved, homogenized, and plated onto M9 agar for enumerating bacterial counts. Based on the KLA infection model established in our previous study [17], groups of two to four thirty-week-old diabetic or naïve mice were orally inoculated with 10^5 ^or 10^8 ^CFU of *K. pneumoniae*, respectively. Twenty microliter of blood was retrieved from the retroorbital sinus of infected mice at 24, 48, and 72 h post-inoculation for enumeration of bacterial counts. Survival of the infected mice was monitored daily for seven days. For histological examination, livers retrieved from mice were fixed in 4% paraformaldehyde, paraffin embedded, and stained with haematoxylin and eosin. All the animal experiments were performed according to NLAC guidance and the Institutional Animal Care and Use Committee approved protocols.

### Statistical analysis

Results are expressed as means ± SD. The two-sample *t *test was used to test for differences between the groups indicated. Statistical significance was determined based on a *P *value ≤ 0.05. All experiments were repeated a minimum of three times to ensure reproducibility.

## Competing interests

The authors declare that they have no competing interests.

## Authors' contributions

YC Lin, HLT and CHC performed the animal studies. HCL, KSL, CL, and CSC made substantial contributions to conception and design, and revised the manuscript critically for important intellectual content. YC Lin, MCL, and YC Lai performed the analysis and interpretation of data. MCL and CMC participated in design and coordination. YC Lin, MKC, and YC Lai drafted the manuscript. All authors read and approved the final manuscript.

## Supplementary Material

Additional file 1 **Induction of diabetic mice**. The file contains supplemental figure S1 that presents the successful induction of diabetic mice in this study. Click here for file
