# Supplementary material for: Assessment of hypermucoviscosity as a virulence factor for experimental Klebsiella pneumoniae infections: comparative virulence analysis with hypermucoviscosity-negative strain
Source: BMC Microbiol. 2011 Mar 8;11:50. doi: 10.1186/1471-2180-11-50 (PMC3060850; doi:10.1186/1471-2180-11-50)
Supplement: Additional file 1 — Induction of diabetic mice. The file contains supplemental figure S1 that presents the successful induction of diabetic mice in this study. [file 1471-2180-11-50-S1.PDF]

## Supplemental Figure

**Figure S1.** Induction of diabetic mice. (A) Blood sugar in diabetic mice (black columns) and age-matched naive mice (white columns) determined at eight and thirty weeks. Data are means ± SD. $^{*}P \leqq 0.05$, significant difference between diabetic and naive mice. $^{\#}P \leqq 0.05$, significant difference on blood levels determined at eight and thirty wk. (B) Body weight in diabetic mice (black columns) and age-matched naive mice (white columns) determined at the indicated time intervals. Data are means ± SD. $^{*}P \leqq 0.05$, with a significant difference between diabetic and naive mice.

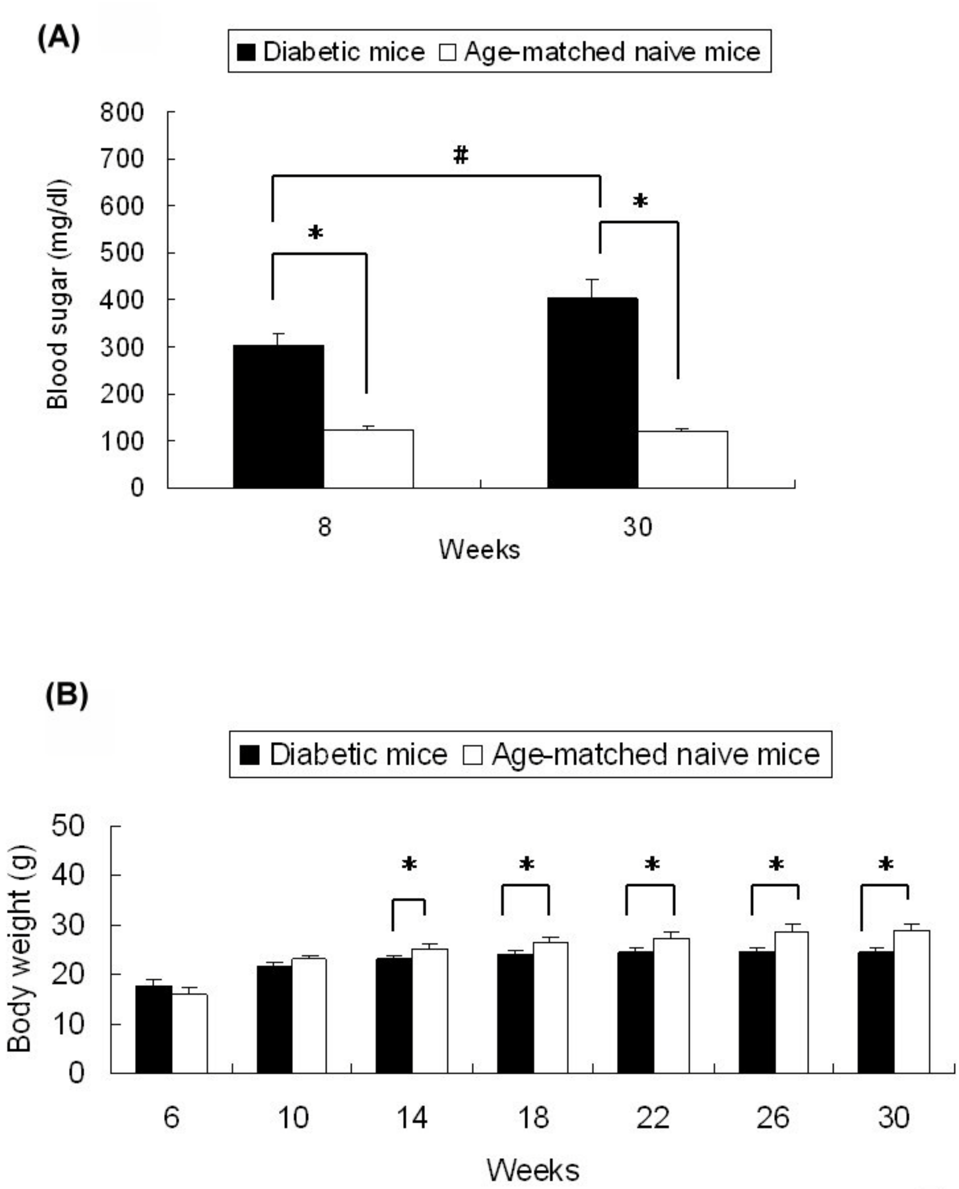


Fig. S1
